# The kinematic profile of ventral swimming start: sex diversity

**DOI:** 10.3389/fphys.2023.1157359

**Published:** 2023-08-01

**Authors:** Daria Malgorzata Rudnik, Marek Rejman, Joao Paulo Vilas-Boas

**Affiliations:** ^1^ Department of Swimming, Faculty of Physical Education and Sport, Wroclaw University of Health and Sport Sciences, Wrocław, Poland; ^2^ Faculty of Sports, University of Porto, Porto, Portugal; ^3^ Center of Research, Training, Innovation and Intervention in Sport, Faculty of Sports, University of Porto, Porto, Porto, Portugal; ^4^ Porto Biomechanics Laboratory, University of Porto, Porto, Portugal

**Keywords:** sex effect, kick-start, performance determinants, kinematics, swimming

## Abstract

It has been suggested that sex distinctions in physiology may affect the swimming performance of each sex differently. Yet, sex-based performance dependency has not been taken into consideration by most of the researchers evaluating swimming start. Therefore, the purpose of this research was to determine the effect of sex heterogeneity on the spatiotemporal characteristics of swimming start by investigating the determinants of its performance. A total of fifty-two international-level swimmers (thirty females and twenty-two males) performed three repetitions of the kick-start up to the 15-m mark. During trials, data were collected using video cameras and instrumented starting block. To search for evidence of differences between the two groups, the one-way ANOVA was conducted. Pearson’s correlation coefficients were calculated between measurements widely used to describe overall starting performance and selected kinematical variables of swimming start. A sex effect was exposed for temporal variables describing all swimming start phases (*p* ≤ 0.015). Male swimmers, by spending less time during the push-off from the starting block (*p* = 0.002; *η*_p_^2^ = 0.18), reaching higher take-off velocity (*p* < 0.001; *η*_p_^2^ = 0.29), traveling longer distances during flight (*p* < 0.001; *η*_p_^2^ = 0.40), and swimming faster in the water phase (*p* < 0.001; *η*_p_^2^ = 0.40), took starting advantage over their female counterparts. Consequently, performance measures such as 5-m, 10-m, and 15-m start times indicated that male participants were faster than females (*p* < 0.001; *η*_p_^2^ ≥ 0.40). Only in the group of male swimmers a significant correlation between variables describing overall starting performance (5-m, 10-m, and 15-m times), and variables commonly highlighted as starting performance determining factors (block phase duration, take-off horizontal velocity, and flight distance) was found. The current study shows that the spatiotemporal variables of swimming start, the relation between them as well as overall starting performance, vary by sex. Consequently, the requirement of sex factor and its heterogeneity effect should be included not only in detailed characteristics of separate variables but also in all approaches undertaken. Those findings seem to play a crucial role mainly in swimming start evaluations in post-pubertal age groups of swimmers.

## 1 Introduction

It has been stated that there is a constant sex gap in sports (Tang et al., 2020). In swimming, success is determined by many factors. It is widely known that sex has a significant impact on sports performance, as physical demands appear to be among the decisive elements in swimming. Moreover, differences between sexes on energetics are related to body mass and composition rather than the profile of contribution and reliance on the energetics components during high-intensity swimming performance (Massini et al., 2021). Yet, the sex variance in the time results of competitive swimming performance became progressively smaller as race distance increases, which could be related to greater reliance on oxygen metabolism (Tanaka and Seals, 1997). That has been attributed to the higher swimming economy of women, as characterized by smaller body size and shorter lower limbs, as well as smaller body density and greater fat percentage (Pendergast et al., 1977; Lavoie and Montpetit, 1986). However, the differences between sexes in swimming performance depend also on the age of the athlete, especially prior to the performance-enhancing effects of puberty (Senefeld et al., 2019). Female swimmers outscored their male counterparts in the case of 10-year-old or younger and older age groups (75 years or more) (Knechtle et al., 2020). Meanwhile, considering other age groups, male swimmers have better overall results in all swimming Olympic events (Miller et al., 1984; Morais et al., 2019).

However, sex-based performance dependency has not been taken under consideration by the researchers who researched in swimming start performance. Many previous studies evaluating swimming start included mixed-sex groups in their analyses (Galbraith et al., 2008; Barlow et al., 2014; Ikeda et al., 2016). In those publications, the lack of significant diversity between the measured variables describing each sex was used as argumentation confirming the methodological approaches chosen by the authors. Other studies reporting start performance within sex groups, did not involve direct comparisons of results between males and females (Jesus et al., 2011; Da Silva et al., 2019; Morais et al., 2019; Arellano et al., 2022) or recruited only a small number of participants (Ruschel et al., 2007; Seifert et al., 2010). Furthermore, in the systematic review of the ventral swimming start, Blanco et al. (2017) took under consideration almost fifty studies, from among which eighteen included both sexes but did not necessarily make a sex division, eighteen of them evaluated males, and only three encompassed females. In ten of those publications, the sex of the participants was not clearly exposed. Then, there is limited research comparing swimming start characteristics in females and males, which confirms the need to search for the sex effect on starting performance.

It is widely known that the anthropometrics and physiological characteristics of the athletes may differently affect the swimming performance of females and males (Senefeld et al., 2016; Rejman et al., 2018). However, sex differences in water activities may be less visible than those during weight-bearing exercise (Senefeld et al., 2016; Tang et al., 2020). Considering ventral swimming start, there is evidence for that, male and female swimmers could undertake different movement patterns to perform similar tasks (Fischer and Kibele, 2014; Fischer and Kibele, 2016). Moreover, concerning sex diversity, differences might exist also in how velocity is developed (Tor et al., 2014). Therefore, results presented by existing studies that combine both sexes in one group may not be considered as valid. Regardless of the participants sex, such variables as block time, take-off horizontal velocity, and flight distance have been widely used by many authors as starting performance indicators (Slawson et al., 2013; Ikeda et al., 2016; Blanco et al., 2017; Morais et al., 2019; de Jesus et al., 2022). That methodologically controversial approach probably arose as a result of analyses including mainly male swimmers or even, in some cases, studies combining both sexes regardless of their potential diversity. Moreover, as simple analyses were based on limited numbers of variables, in specific instances, the mentioned differentiation might not be exposed, especially when the sample size was also reduced. It is important to underline that swimming performance depends on many biomechanically determinant factors (e.g. anthropometrics, kinematics, hydrodynamics) and relationships between them (Morais et al., 2013). Concluding, in the context of the current knowledge review, the multifaceted comparative analysis describing kick-start performed by swimmers of both sexes seem to be needed.

Consequently, this study aimed to explore sex diversity with regard to the variability of the spatiotemporal variables of the kick-start technique executed by international-level swimmers. Besides, the purpose of this research was to determine the effect of sex heterogeneity on the biomechanical characteristics of swimming start by investigating the determinants of its performance. The findings could indicate the variables that should be considered in an objective assessment of swimming start performed by males and females separately.

## 2 Materials and methods

A total of fifty-two swimmers, comprising thirty females (16.9 ± 2.2 years of age, 168.9 ± 4.4 cm of body height, and 59.3 ± 4.7 kg of body mass) and twenty-two males (18.3 ± 1.8 years of age, 178.9 ± 5.3 cm of body height, and 69.9 ± 5.9 kg of body mass) volunteered to participate in the study. A swimmer was considered at an international level when was a member of the national swimming team and held a personal record at the level of at least 750 FINA points. Before signing an institutionally established informed consent, all swimmers and their coaches were informed about the purpose of the study and the testing protocol. The study followed the tenets of the Declaration of Helsinki and was approved by the local Ethical Board.

In the initial part of the experimental session, the athletes performed their conventional pre-race warm-up routine and had time to familiarize themselves with the instrumented starting block equipped with measurement devices (Vilas Boas et al., 2014; Vitor et al., 2016). The swimmers were asked to simulate a 20-m sprint race after the kick-start. They were encouraged to simulate the race behavior, i.e. to achieve the shortest possible time of the trial distance (measured between the starting signal and the instant when the swimmer head reached the marker at 15-m from the starting platform. To keep the highest possible performance for the next trial, at least 3 minutes of a passive resting break was provided between all repetitions. Each swimmer performed three repetitions of the kick-starts. The best trial—considering the 15-m time as the main performance predictor—was chosen for further analyses.

**FIGURE 1:**
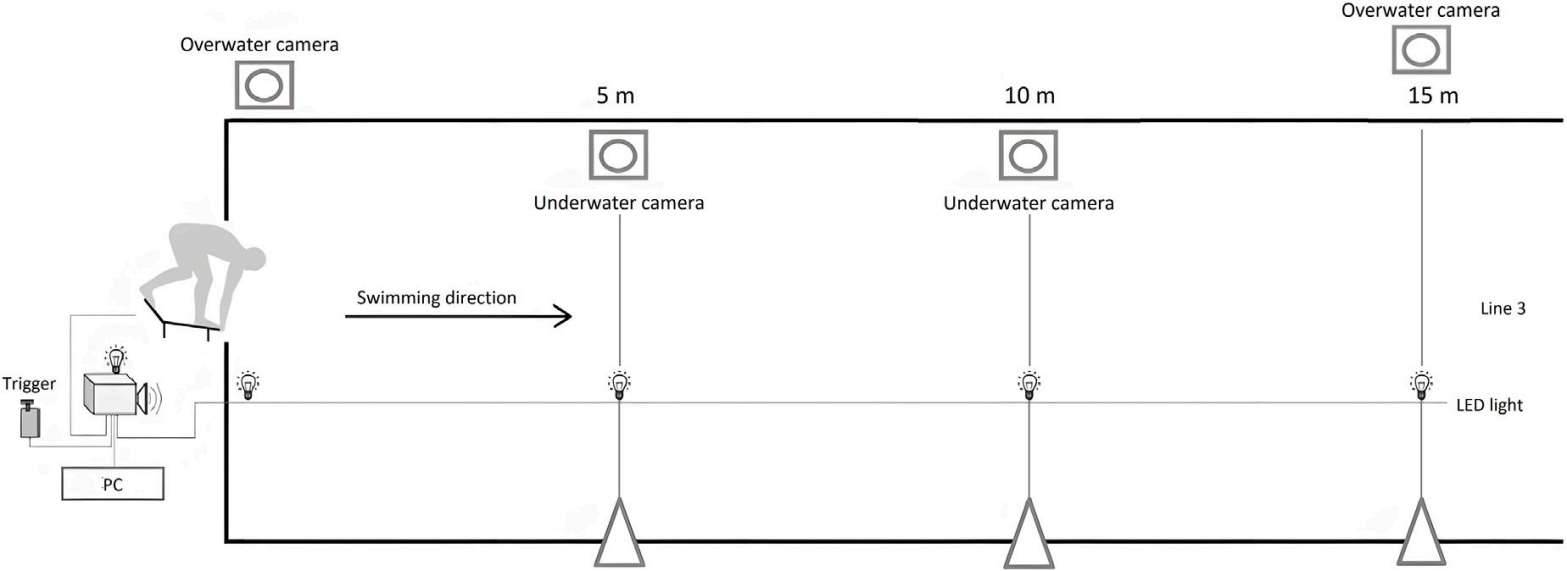
Graphical presentation of the measurement equipment setup.

Figure 1 illustrates the equipment setup used during the experiment. The 2D kinematic setup consisted of four video cameras (HDR CX160E, Sony Electronics Inc., Japan, and GoPro Hero 4, GoPro, United States), placed on the side of the swimmer, with their optical axis perpendicular to the swimming start trajectory. The videotaping frequency was adjusted to 50 frames per second, with a resolution of 1920 × 1,080. Two surface video cameras were fixed to tripods (Hama Star 63, Hama Ltd., United Kingdom) at a height of 0.75 m. The first one was dedicated to capturing the swimmers’ movements from the starting signal until total immersion of the swimmer body; the other one was used for a 15-m start time measurement. Two underwater video cameras, located on the sidewall of the pool, were applied to record the swimmers while passing the markers located 5-m and 10-m from the starting line. All the cameras were calibrated with a 2 × 2-m frame and synchronized with the LED light, visible in each of the cameras.

The starting signal was given by the starting device (Onda TTL wave, 0–5 V), which acted as a trigger providing simultaneous sound, visual, and electrical signals, supporting synchronization of all equipment used for data acquisition (Vitor et al., 2016). To obtain a higher sensibility of temporal data during the block phase, a 3D-6DoF instrumented starting block compliant with the current World Aquatics regulations and replicating OMEGA OSB 14 was used. The athletes were dressed in textile swimsuits and had 32 landmark points marked on their bodies (Juergens, 1994), which allowed to define their body parts displacement in a two-dimensional plane.

The raw data were synchronized with the starting signal and processed with the use of dedicated software. To expose the spatiotemporal characteristics of the start, the SIMI Motion System (SIMI Reality Motion Systems GmbH, Germany) was applied for manual digitization and video image data treatment. Independently, a processing routine created in the MATLAB R2016a software (MathWorks Incorporated, United States) was employed to derive the temporal characteristics of the block phase on the basis of data collected with the instrumented starting block. The variables selected for further analysis are described in Table 1. These variables are also included on a regular basis in studies evaluating swimming starts (Tor et al., 2014; de Jesus et al., 2022; Rudnik et al., 2022).

**TABLE 1:** Definitions of the variables used to characterize the structure of the swimming start.

Variable	Definition
Reaction time (s)	The time interval between the starting signal and a change in starting block reaction force curve as a result of the initial movement
Hands take-off (s)	The time interval between the starting signal and the last contact of the hands with the starting block
Rear foot take-off (s)	The time interval between the starting signal and the last contact of the rear foot with the starting block
Front foot support (s)	The time interval between the last contact of the rear foot with the starting block and the moment when total vertical force fell to zero
Block time (s)	The time interval from the starting signal and the moment when total vertical force fell to zero
Movement time (s)	The time interval from the first visible change in starting block reaction force curve and the instant when total vertical force fell to zero
Take-off horizontal velocity (m/s)	The instantaneous horizontal velocity of the swimmer measured at the moment of take-off
Flight time (s)	The time interval between the last contact of the toes with the block and the moment of first contact of the hands with the water
Flight distance (m)	The horizontal distance measured between the point where the hip entered the water and the starting line
Take-off angle (°)	The angle between the horizontal axis, the block edge, and the hip joint at take-off
Entry angle (°)	The angle between the horizontal axis, the fingertips, and the hip joint when hands entered the water
5-m time (s)	The time interval between the starting signal and the moment when the head crossed the 5-m mark
5–10 m time (s)	The time interval between the moment when the head crossed the 5-m mark and the moment when the head reached the 10 m distance from the starting line
10-m time (s)	The time interval between the starting signal and the moment when the head crossed the 10-m mark
10–15 m time (s)	The time interval between the moment when head crossed the 10-m mark and the moment when the head reached the 15-m distance from the starting line
15-m time (s)	The time interval between the starting signal and the moment when the head crossed the 15-m mark
5–15 m average velocity (m/s)	The average swimmer velocity the between the 5-m and 15-m marks

The selected variables describing spatiotemporal movement characteristics were used for further analyses, and results were scrutinized to expose any significant differences among values of variables representing different sexes. Furthermore, to screen if sets of data did not include any extraneous or confounding variables, as well as represented normal distributions and homogeneity of variance, the Shapiro-Wilk test, Levene’s test, and descriptive statistics were calculated for each variable. Concerning the above, the assumptions of the parametric tests were confirmed, and the values of the variables were presented as means and standard deviations. Accordingly, a one-way analysis of variance for independent samples was conducted, allowing statistical inference whether there was statistically significant evidence of a difference between two unrelated groups. To argument the significance of the tests, the effect size was calculated and reported. The effect size was exposed with partial eta squared (*η*_p_^2^). The Statistica 13.1 software (StatSoft, United States of America) was applied for all statistical computations (*α* = 0.05).

## 3 Results

The values of the selected spatiotemporal variables taken under consideration are described as means and standard deviations and presented in Table 2. Generally, the male group achieved relatively better results than the females. Males achieved shorter times for 5-m (less by ca. 0.18 s), 10-m (less by ca. 0.51 s), and 15-m (less by ca. 0.72 s) distances. Looking at the sex differences in the time results for each 5-m segment from the start, the highest diversity was measured for the time of the 5–10 m distance. The difference in gap time between the male and female participants increased continuously with the starting distance. Male swimmers spent less time (ca. 0.029 s) on the block (*p* = 0.015). Their advantage was also in higher values of take-off horizontal velocity (more by ca. 0.28 m/s) and flight distance (more by 0.3 m). It can be noticed that the groups did not differ considering spatial variables as take-off or entry angles (*p* > 0.05).

**TABLE 2:** Descriptive statistics for spatiotemporal variables of the swimming start, presented by sex, and between-sex comparisons obtained with oneway ANOVA.

Phase		FEMALE	MALE	ANOVA		
	Variable	Mean ± SD	Mean ± SD	F	p	η_p_^2^
Block	Reaction time	0.161 ± 0.03	0.167 ± 0.03	0.57	.453	0.01
	Hands take-off	0.440 ± 0.11	0.453 ± 0.08	0.23	.631	0.00
	Rear foot take-off	0.620 ± 0.04	0.599 ± 0.05	2.90	.095	0.05
	Front foot support	0.131 ± 0.02	0.127 ± 0.02	0.36	.552	0.01
	Block time	0.745 ± 0.04	0.716 ± 0.05	6.30	.015*	0.11
	Movement time	0.584 ± 0.03	0.548 ± 0.05	10.62	.002*	0.18
	Take-off horizontal velocity	4.096 ± 0.21	4.372 ± 0.23	19.81	<.001*	0.29
Flight	Flight time	0.253 ± 0.05	0.288 ± 0.04	6.91	.011*	0.12
	Flight distance	2.533 ± 0.17	2.834 ± 0.20	33.59	<.001*	0.40
	Take-off angle	32.3 ± 4.7	33.8 ± 4.4	1.35	.251	0.03
	Entry angle	38.9 ± 3.8	37.3 ± 4.0	2.44	.125	0.05
Water	5-m time	1.705 ± 0.09	1.529 ± 0.12	33.76	<.001*	0.43
	5–10 m time	2.681 ± 0.22	2.352 ± 0.18	29.51	<.001*	0.40
	10–15 m time	2.780 ± 0.24	2.557 ± 0.23	10.19	.003*	0.19
	15-m time	7.128 ± 0.34	6.410 ± 0.45	42.95	<.001*	0.46
	Vx 5-15 m	1.837 ± 0.11	2.048 ± 0.16	29.50	<.001*	0.40

Vx 5–15 m (average horizontal velocity calculated using the formula distance over time during 5–15 m *Significant at exact *p* ≤ 0.05.

**FIGURE 2:**
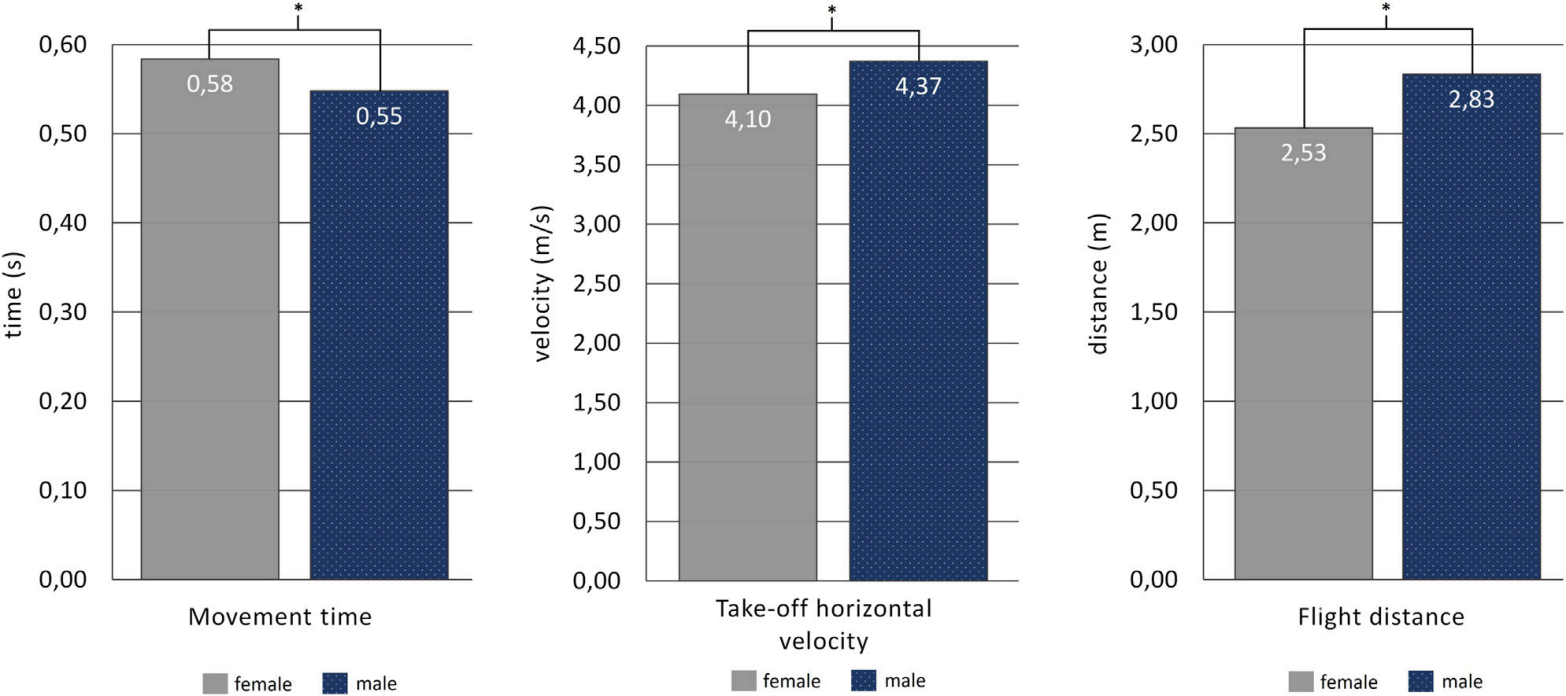
Movement time, take-off horizontal velocity, and flight distance results compared between sexes.

Male athletes needed a shorter time to propel themselves from the starting block, even though they reached higher values of the take-off horizontal velocity and displaced their bodies further overwater in forward directions (Figure 2). The results imply that sex had a significant effect on the measured spatiotemporal variables that describe the kick-start structure and its consequences up to 15-m distance from the starting line (Table 2; Figure 2).

Pearson correlation coefficients calculated for males and females between several time periods of the swimming start and its selected spatiotemporal variables are presented in Table 3. In the male group, higher values of the swimming start time were correlated with take-off horizontal velocity (r <—0.48), flight distance (r <—0.50), and block phase duration (r > 0.41). While those relationship has not been observed for the female group of swimmers. A sex effect was also observed for selected variables of starting performance determinants (block phase duration, take-off horizontal velocity, and flight distance). A general overview of the aforementioned correlation analysis shows that starting performance measured in a wide range of distances exposed sex diversity in relation to selected spatiotemporal variables of swimming start.

**TABLE 3:** Pearson correlation coefficients estimated between several time intervals of swimming start and selected spatiotemporal variables, by sex.

	5-M time		5–10 m time		10-M time		10–15 m time		15-M time	
	M	F	M	F	M	F	M	F	M	F
Take-off horizontal velocity	–0.69*	–0.27	–0.48*	–0.18	–0.65*	–0.26	–0.50*	–0.20	–0.66*	–0.34
Flight distance	–0.55*	–0.16	–0.64*	–0.16	–0.69*	–0.20	–0.50*	–0.03	–0.69*	–0.17
Block time	0.52*	0.36	0.41	–0.35	0.52*	–0.18	0.45	–0.02	0.56*	–0.15
Flight time	–0.17	0.09	–0.29	0.04	–0.28	0.07	–0.25	0.05	–0.30	0.09

M: males; F: females. *Significant at exact *p* ≤ 0.05.

## 4 Discussion

### 4.1 General trends

To determine whether sex diversity resulted with regard to the variability of the spatiotemporal variables of the kick-start movement pattern, the ANOVA analysis was conducted, demonstrating some significant and strong effects of the sex factor. The results pointed out multiple differences in swimming start characteristics and performance between female and male swimmers (Table 2). In general, these results were in line with several studies assessing swimming start performance. According to Newton (2014), there is a clear presence of differences in strength, performance, and technical characteristics between male and female swimmers. Here, as implied by Senefeld et al. (2019), sex distinctions in physiology (e.g. more subcutaneous fat, body size, limb length, and body density) may affect the swimming performance of each sex differently. Therefore. Combining both sexes in the same analysis may not be appropriate. Consequently, the requirement of sex factor and its heterogeneity effect should be included not only in the detailed characteristics of separate variables but also in all approaches undertaken.

### 4.2 Comparison of swimming start characteristics between sexes

The results obtained in the current study exposed no significant differences between sexes in the reaction time (Table 2). Based on data obtained with the Cybex Reactor from the group of college athletes, Spierer et al. (2010) exposed sex effect on reaction time only while an auditory signal was provided. While regardless of the stimuli, in men movement time was reduced as compared to women. According to those authors, sex differences may be influenced by the number of muscle fibers activated for movement. If any sex differences in reaction time exist, still they are attributed largely to inherent as related to the speed of information processing (Adam et al., 1999).

In the current study, the total start time (15-m), the duration its shares parts (5-m, 10-m), and almost all of the time periods of starting phases were shorter for the male group (Table 2). This is in line with the majority of studies presenting less block time and start time for male than female swimmers (Jesus et al., 2011; Thanopoulos et al., 2012; Garcia-Hermoso et al., 2013; Tor et al., 2014; Morais et al., 2019; Arellano et al., 2022; Rudnik et al., 2022). Obtained results shown that, the female swimmers spent ca. 0.029 s more on the block than their male counterparts, who needed 0.716 s to push off from the starting platform (Table 2). Slawson et al. (2013) found block time values in the range of 0.735–0.865 s for 19 females and 0.726–0.856 s for 27 males. A comparison of block time and the final results of world championships events (for 45 females and 57 males) delivered by Da Silva et al. (2019) exposed similar trends in both sexes. Those results were obtained among presented mean block time values of ca. 0.64–0.71 s and 0.62–0.71 s, respectively. It is also in line with the results presented by Arellano et al. (2022). In contrast, Fischer and Kibele (2016) showed equal mean grab-start block time and shorter mean track-start block time for female swimmers. Despite this, in general, male swimmers seem to need less time to push-off from the starting block while starting.

Considering flight time and flight distance, lower values were obtained in the female group (Table 2) A shorter flight time for females was also measured in the study by Thanopoulos et al. (2012) (0.41 ± 0.07 s vs 0.38 ± 0.06 s). It was a result of a longer flight distance obtained by males (3.14 ± 0.20 m vs 2.73 ± 0.21 m). Also, Arellano et al. (2022) presented a shorter time and distance of the flight phase for males. In the mentioned study, the gap differed depending on the round of the individual competitive event taking place during the 2021 European Championships. That pattern was also observed in the study by Morais et al. (2019), who showed longer for ca. 0.04 s flight time and for ca. 0.38 m flight distance for males compering to females in a freestyle event. Yet, according to Ruschel et al. (2007), flight time is less significant than flight distance as a starting performance determining factor.

Male swimmers obtained a higher take-off velocity and displaced their hips further during the flight phase (Figure 2), which is also consistent with the findings by Slawson et al. (2013). According to those authors, the block time, take-off horizontal velocity, and flight distance were among the main indicators of swimming start performance. In the quoted study, a methodology for categorizing swimming start performance was based on the peak force data analyses. Here, the peak forces produced by females were lower than those in male athletes. In general, it is reasoned by the higher muscle power leading to an improvement in the block start impulse in male swimmers (Jesus et al., 2011). Similar results were obtained by Tor et al. (2014), who characterized the start of elite swimmers, including a comparison of start variables and their diversity between the sexes. From all variables considered in the evaluation of swimmers’ overwater actions, only take-off vertical velocity and flight time did not differ between sexes (Tor et al., 2014). The consequences brought by the presented results have been further confirmed by Rudnik et al. (2022) who examined the impact of back plate position on the characteristics of the kick-start. Here, changes in the back plate position affected more male than female swimmers.

Data reported by Fischer and Kibele et al. (2014) provide evidence that male and female swimmers undertake different movement patterns to perform similar tasks during starts. As a result, the variables describing movement structures of the entry phase (average horizontal velocity, the angular displacement of the hip joint, and the duration of the entry phase) varied significantly between sexes. Therefore, the same authors, focusing mainly on grab-start and track-start comparison, showed that sex diversity was expressed for even more specific variables, including vertical take-off velocity and relative height at take-off (Fischer and Kibele, 2016), which determine flight and water entry profiles. Also, in the referred study, the flight and water phases were different between males and females. These results are coherent with those suggesting different technical underwater strategies undertaken by swimmers of each sex (Tor et al., 2014). Here, males swam longer and deeper underwater. Besides, timing and velocity values measured from 5-m up to 15-m in that study corroborate our findings. Regardless of the higher level (in terms of the mean time values) of swimmers evaluated by Tor et al. (2014), the quoted results revealed a profile of diversity similar to our observations. Considering that male swimmers benefit from shorter block time, higher take-off horizontal velocity, and longer flight distance, thus they are able to successfully transfer the energy included in those phases into underwater gliding.

### 4.3 Factors determining starting performance

Generally, there is a trend that males present a shorter block time, longer flight distance, and higher horizontal velocities through the start which consequently ensures them shorter total start time. At the same time, in the current study depending on the sex of the swimmer, those variables relate differently to the total start time (Table 3). Besides, only for males a significant correlation between overall starting performance and block time, take-off horizontal velocity, and flight distance was presented. On the contrary, the Pearson product-momentum correlation coefficients expose low values of these variables for women. Meanwhile, those variables have been widely used as starting performance indicators regarding the sex of the swimmers (Slawson et al., 2013; Ikeda et al., 2016; Morais et al., 2019). Yet, the correlations between the same variables observed previously by Garcia-Ramos et al. (2015) did not confirm the results obtained for the female group (Table 3). The current findings thus demonstrate the need for sex separation in the assessment of start performance based on factors selected as significantly relating to it.

Morais et al. (2022) while searching for predictors of swimming velocity, obtained results not only exposing an interplay of variables related to anthropometry, kinematics, and kinetics but also confirmed our findings by revealing a significant sex effect. It is widely known that the anthropometric profiles of athletes might have a significant influence on swimming performance (Rejman et al., 2018; Alves et al., 2022). Similar conclusions can be drawn when assessing starting performance, but here, sex-related anthropometrics would determine the results in a different way. Male swimmers would take advantage of body mass, body height, muscle strength, and power, which are crucial in the push-off phase and its consequences. On the other hand, females would compensate for their lower profile in these variables through the more hydrodynamic body shape and body density, which gain special relevance during the water phase. Vantorre et al. (2010) suggested that better gliding performance was attributed to a slimmer body, as taking advantage of better hydrodynamics. However, while looking at the undulatory propulsion movement in the underwater phase, the power of propulsion overtakes the benefits of the body shape. Indeed, following Tor et al. (2014), the description of spatial underwater phase characteristics shows lower sex dependence than the block phase. While for final events on 50 m freestyle, Arellano et al. (2022) showed lower for ca. 0.34 m/s underwater speed for female swimmers comparing to their male counterparts. In short, not only overall race performance but also the starting phases are related to the anthropometrical gap based on sex diversity (Jesus et al., 2011). In the light of presented findings, while considering the obtained results through the prism of a link between anthropometrics and hydrodynamics the need to differentiate variables employed to evaluate the swimming start performance focusing on the athlete sex become more justified.

Here considering the fact that the swimming start is a sum of phases that are characterized by specific movement patterns. Thus, the size of the sex gap should differ depending on the phase of the start. It seems that men outperform women in sports that require muscular strength or endurance. Considering whole swimming races, the percentage difference between sexes accounted for ca. 7%–11%, while for the total start time it was ca. 14.4% (calculated afte McClelland and Weyand, 2020). Findings from other sports confirm the sex skill gap existence (Tang et al., 2020). For example, running has a significantly smaller disparity between the sexes than jumping does (Carroll, 2019). Indeed, mean male/female differences across jumping events (17.8 ± 2.7%) were 1.5 times greater than those presented for running events (11.2 ± 1.4%) (McClelland and Weyand, 2020). Meanwhile, for canoeing, it has been revealed that the inter-item correlation of performance measures is influenced not only by the sex but also by the age of the athlete (Saal et al., 2022). Finally, it is important to be aware that the sex gap in sports varies also with performance level (Hallam and Amorim, 2022).

## 5 Conclusion

The study confirms that the spatiotemporal variables of the swimming start, the relationships between them, as well as the overall starting performance, differ between sexes. Here, such performance variables as 5-m, 10-m, and 15-m start times indicate that male participants were faster than females. In general, temporal movement organization during the block sub-phases did not differ between the two groups. Yet, male swimmers, by spending less time in the block phase, reaching higher take-off velocity, obtaining longer flight distance, and swimming faster while in the water, took a starting advantage over their female counterparts.

Correlations between separate variables and main variables in the assessment of overall swimming start performance were also presented as varying between the two groups. Variables commonly used for swimming start performance assessment, such as take-off velocity, flight distance, or block time duration, correlated significantly with overall start performance only in the group of male athletes. In light of this finding, it is important to differentiate variables employed to evaluate the swimming start performance considering the sex of the athlete.

The findings seem to play a crucial role in swimming start sex evaluation in post-pubertal age groups of swimmers. Therefore, as we aim to contribute to knowledge development and, consequently, to support swimmers and their coaching staff in the starting performance enhancement, the study outcome should attract considerable attention among practitioners. The presented findings could contribute to future practice, clarifying which variables should be considered while objectively evaluating start performance in male and female swimmers.

Notwithstanding the relevance of the undertaken approach, some limitations resulting from the methodology of this research should be addressed. The first one concerns the free choice of the starting technique applied by the participants, which was based on their previous experience and specified for each subject independently. Consequently, the starting position could have influenced the spatiotemporal characteristics of the start. Therefore, in the context of the presented conclusions, a more detailed analysis of underwater actions should shed more light on the sex effect while starting. Further research is needed to explore such factors as performance level, biomechanical demands, anthropometrics, specific motor abilities, and relationships between them, to exhaustively describe the crucial variables determining the swimming start performance considering the sex of the swimmer.

## Data Availability

The original contributions presented in the study are included in the article/Supplementary Material, further inquiries can be directed to the corresponding author.
